# The Relation between eNOS −786 C/T, 4 a/b, MMP-13 rs640198 G/T, Eotaxin 426 C/T, −384 A/G, and 67 G/A Polymorphisms and Long-Term Outcome in Patients with Coronary Artery Disease

**DOI:** 10.1155/2015/232048

**Published:** 2015-09-30

**Authors:** Vladimír Kincl, Jan Máchal, Adéla Drozdová, Roman Panovský, Anna Vašků

**Affiliations:** ^1^Department of Internal Medicine/Cardioangiology and ICRC, St. Anne's University Hospital, Faculty of Medicine, Masaryk University, Pekařská 53, 656 91 Brno, Czech Republic; ^2^Department of Pathological Physiology, Faculty of Medicine, Masaryk University, Kamenice 5, 625 00 Brno, Czech Republic

## Abstract

*Aim*. The purpose of this study is to determine the association between eotaxin 426 C/T, −384 A/G, 67 G/A, eNOS −786 T/C, 4 a/b, and MMP-13 rs640198 G/T and prognosis of patients with known CAD.* Methods*. From total of 1161 patients referred to coronary angiography, 532 patients with angiographically confirmed CAD were selected. Their long-term outcome was followed up using hospital database. Subsequent events were assessed in this study: death or combined endpoint-myocardial infarction, unstable angina pectoris, revascularization, heart failure hospitalization, and cardioverter-defibrillator implantation.* Results*. The multivariate Cox regression model identified age, smoking, and 3-vessel disease as significant predictors of all-cause death. Further analysis showed that eotaxin 67 G/A (GA + AA versus GG) and eotaxin −384 A/G (GG versus GA + AA) were significant independent prognostic factors when added into the model: HR (95% CI) 2.81 (1.35–5.85), *p* = 0.006; HR (95% CI) 2.63 (1.19–5.83), *p* = 0.017; eotaxin −384 A/G was significantly associated with the event-free survival, but it did not provide the prognostic information above the effect of two- or three-vessel disease.* Conclusion*. The A allele in eotaxin 67 G/A polymorphism is associated with worse survival in CAD patients.

## 1. Introduction

Cardiovascular diseases, particularly coronary artery disease (CAD), are the leading cause of mortality in developed countries. The aetiology of CAD is multifactorial and various well-known risk factors have been described in previous decades. Association of CAD prevalence and deoxyribonucleic acid (DNA) polymorphisms in human genes has been extensively studied [1, 2], but relation of these polymorphisms to long-term prognosis of patients was reported less frequently [3–6]. Some of the polymorphisms did not show any significant association with the prognosis of CAD or the results were controversial. On the other hand, several papers reported promising results; for example, genome-wide association study (GWAS) identified polymorphisms in loci 1p13.3 (rs599839), 1q41 (rs17465637), and 3q22.3 (rs9818870) [7], or the variants of paraoxonase 1 gene (rs854560) [5] and phospholipase A2 gene (rs1805017) [6]. These data were repetitively confirmed by following studies [8–14]. However, there are many variants in human genome influencing the presence of CAD, of which their relationship to long-term outcome in CAD patients is still not elucidated as the information based on prospective studies is lacking.

Matrix metalloproteinases are zinc-dependent endopeptidases cleaving proteins of extracellular matrix [15]. Matrix-metalloproteinase 13 (MMP-13) preferentially cleaves type II collagen and is produced by macrophages and smooth muscle cells in atherosclerotic lesions [16]. This contributes to restructuring of atherosclerotic plaque in animal models [17]. Polymorphisms of MMP-13 gene influencing its production have been associated with an extent of atherosclerosis [18], including rs640198 G/T intronic single nucleotide polymorphism (SNP) [19].

Eotaxin (CCL11) is a chemokine with pleiotropic proinflammatory effects produced by several cellular types present in atherosclerotic lesion [20]. Studies in human subjects have found eotaxin overexpression in atherosclerotic plaques [16] or its elevated plasmatic concentration in the presence of coronary atherosclerosis [21, 22]. Genetic variation of eotaxin gene, SCYA11, has been found to contribute to CAD susceptibility, most notably the first exon (67 G/A) polymorphism leading into amino acid change Ala/Thr in site 23, where secreted eotaxin is cleaved off from the signalling peptide [23]. The association of eotaxin (CCL 11) Ala/Ala variant and development of coronary artery disease was reported by Wang et al. [24]. The authors performed a prospective study of 1297 Chinese patients with diabetes mellitus; median follow-up was 8 years. The variant Ala/Ala of eotaxin Ala23Thr polymorphism showed a hazard ratio of 1.7 (95% CI 1.10–2.61, *p* = 0.037). Other functionally relevant polymorphisms were identified in the promoter of the gene, as −384 A/G and −426 C/T [23], which has been associated with eotaxin levels and asthma phenotype [25, 26].

Endothelial nitric oxide synthase (eNOS) is an enzyme that constitutively produces nitric oxide from L-arginine, mediating vasodilation and having other beneficial effects on vessel wall [27]. The rate of NO synthesis by eNOS is modulated by variability in eNOS gene, which is the case of 27-bp tandem repetition in intron 4, where an “A allele” (consisting of four repetitions) is associated with higher NO production than a “B allele” (consisting of five repetitions) [28]. Other polymorphisms, −786 T/C promoter SNP, have been linked to changes in eNOS promoter activity and coronary spasms [29].

The aim of our study is to assess the relationship between polymorphisms in eotaxin gene (67 G/A, −384 A/G, and −426 C/T), MMP- 13 gene (rs640198), and endothelial NO-synthase (eNOS) gene (−786 C/T and 4 a/b) and a long-term outcome in Caucasian patients initially referred to coronary angiography.

## 2. Patients and Methods

### 2.1. Patient Cohort and Genetic Analyses

The study comprised of 532 subjects suffering of CAD that were recruited from the total number of 1161 consecutive patients referred to coronary angiography after applying the following exclusion criteria: known malignancy, advanced renal dysfunction (blood creatinine level above 200 *μ*mol/L), smooth coronary arteries or insignificant atherosclerosis, or life expectancy below 1 year. All patients gave their informed consent and study was approved by the institutional ethic committee. The personal and family history were obtained and all patients underwent physical examination, electrocardiography, haematological and biochemical blood tests, and coronary angiography. Coronary artery disease was defined as ≥50% stenosis of at least one coronary artery. Blood samples for DNA analysis from peripheral blood leucocytes were also taken. The selection of DNA polymorphism, analyses, results, and impact on incidence and severity of CAD were described in our previous publications [19, 30, 31].

### 2.2. Follow-Up

The clinical outcome of patients was assessed in 2014 using hospital database. The following events were recorded: death, acute myocardial infarction (MI), unstable angina pectoris, necessity of myocardial revascularization (either percutaneous or coronary artery bypass graft), hospitalization for heart failure, and cardioverter/defibrillator (ICD) implantation. Patients who were not under clinical observation in our hospital, patients without angiographically confirmed significant CAD, or those with unsuccessful DNA analysis were excluded from the study.

### 2.3. Statistical Analysis

Multivariate Cox regression model was used for estimating the contribution of genetic polymorphisms and other risk factors to survival. Genetic variants had been preselected out of the total number of 8 polymorphisms in candidate genes using Kaplan-Meier method and log-rank tests in dominant, recessive, and codominant mode of allelic expression. *p* value 0.05 was used as a cut-off for an inclusion of variable in the Cox regression analysis. Hardy-Weinberg equilibrium was calculated for each polymorphism using *χ* ^2^ test. Nongenetic variables included in the model were the following: age on admission, sex, presence of acute coronary syndrome, MI in personal history, ejection fraction, number of diseased vessels, obesity, hypertension, hyperlipidaemia, diabetes mellitus, concomitant presence of cardiomyopathy, significant valvular disease, and smoking status. Two endpoints were observed: all-cause death and cardiac endpoint consisting of MI, unstable angina, coronary revascularization, and rehospitalization for heart failure. Supposing unknown dominance of alleles, three modes of gene expressions were used: dominant, where minor allele carriers were compared with major allele homozygotes; recessive, where major allele carriers were compared with minor allele homozygotes; and additive, gene dose-based model, where the values 0, 1, and 2 were attributed to major allele homozygote, heterozygote, and minor allele homozygote. Stepwise construction of a Cox regression model with *p* value to include 0.05 and *p* value to remove 0.051 was finally employed to determine all significant independent factors (clinical and genetic) contributing to patients' survival or the incidence of cardiac event. STATISTICA software (StatSoft, version 12) was used for statistical analysis. MIDAS software version 1.0 [32] was used for Hardy-Weinberg and linkage disequilibrium determination. Generally, *p* values ≤ 0.05 were considered to be statistically significant.

## 3. Results

**Figure 1:**
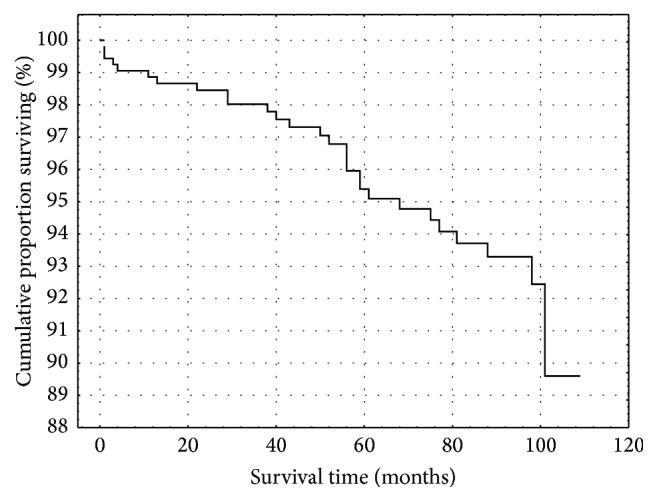
Kaplan-Meier analysis of survival in patients cohort.

**Figure 2:**
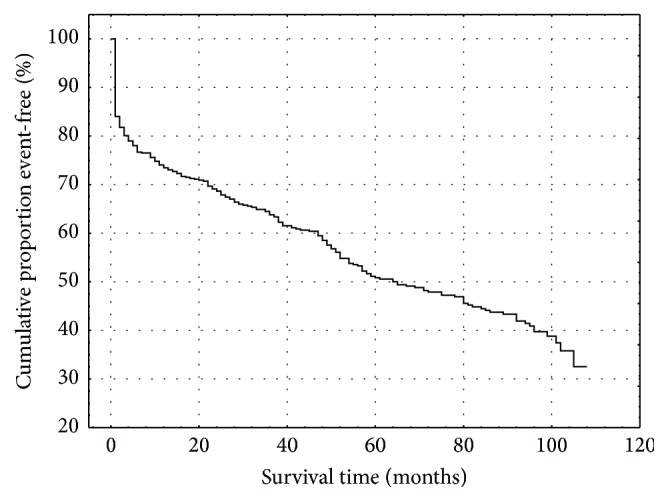
Kaplan-Meier analysis of event-free survival in patients cohort.

Five hundred thirty-two patients with significant CAD were finally comprised into analysis. The basic characteristics are listed in Table 1. Twenty-nine patients died and 271 cardiac events were recorded (26 patients with unstable angina, 44 acute MI, 154 myocardial revascularization, 26 hospitalization for heart failure, and 21 ICD implantation) during follow-up, median follow-up period was 77 months. Kaplan-Meier curves presenting proportion of survival and event-free survival can be seen in Figures 1 and 2. The list of studied polymorphisms and their genotype distribution are shown in Table 2. The preselection of candidate polymorphisms was based on log-rank *p* values. The eNOS 4 a/b, eotaxin −426 C/T, eotaxin −384 A/G, and eotaxin 67 G/A were found to be significantly associated with death, as well as eotaxin −384 A/G with cardiac events. There was also nonsignificant trend for eotaxin 67 G/A to influence the event-free survival.

**Figure 3:**
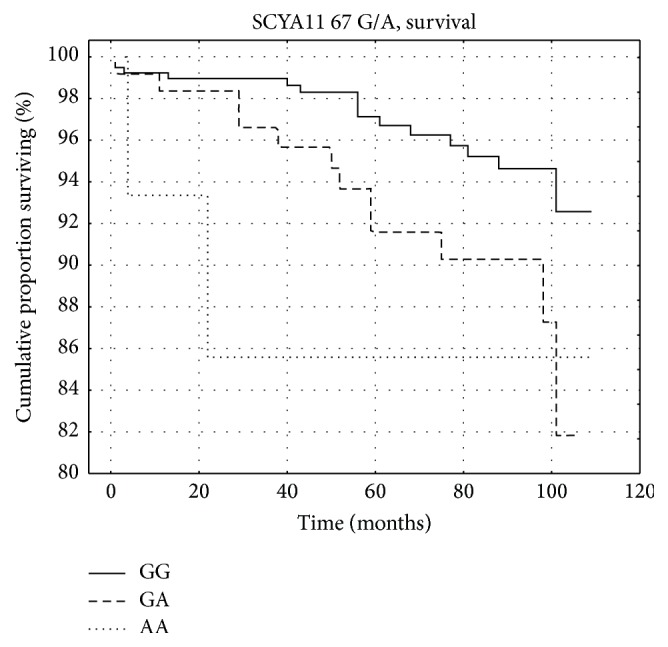
Kaplan-Meier analysis of survival by 67 G/A genotype.

Complete data are shown in Tables 3 and 4. Subsequently, the multivariate Cox regression model was used to reveal potential effects of selected variants on survival and cardiac endpoint-free survival. The eotaxin 67 G/A and eotaxin −384 A/G were considered significant for survival and event-free survival, respectively (Tables 5 and 6). The multivariate stepwise Cox regression model identified age, smoking, and three-vessel disease (compared to one vessel disease) as significant predictors of all-cause death. Together with those factors, eotaxin 67 G/A was a significant independent prognostic factor when added into the model (Table 7, Figure 3). When eotaxin −384 A/G was added into the stepwise Cox regression model instead of 67 G/A, the model gave similar results with the same prognostic factors; however, hazard ratio for the given polymorphism was slightly lower (GG versus GA + AA; HR = 2.63; 95% CI = 1.19–5.83; *p* = 0.017). As shown below, both polymorphisms were in significant linkage disequilibrium, and the “risky” alleles of both SNPs were frequently inherited together. In the multivariate stepwise Cox regression analysis of cardiac endpoints, a number of diseased coronary arteries (two versus one: HR = 1.86; 95% CI = 1.23–2.79; *p* = 0.003, three versus one: HR = 4.80; 95% CI = 3.32–6.94; *p* < 0.001) was the only significant independent risk factor. Adding eotaxin −384 A/G into the model did not change the results; that is, although the −384 A/G polymorphism was associated with the hazard of cardiac endpoint both with and without adjustments, it does not provide the prognostic information above the effect of two- or three-vessel disease. Finally, it was determined, whether the variants in eotaxin and eNOS gene were or were not in linkage equilibrium. The analysis revealed modest, but significant linkage disequilibrium (LD) between polymorphisms −426 C/T and −384 A/G in eotaxin gene promoter (*D*′ = 0.43; *r* ^2^ = 0.02; *p* = 0.01), as well as between −384 A/G and 67 G/A in the same gene (*D*′ = 0.69; *r* ^2^ = 0.09; *p* = 10^−10^). In this polymorphism, the risky A allele of 67 G/A was more often inherited together with the risky G allele of 384 A/G, suggesting the possible role of LD in resulting effects. On the other hand, −426 C/T and 67 G/A were in linkage equilibrium with each other. In eNOS gene, the eNOS −786 C/T and eNOS 4 a/b polymorphisms were also in significant LD with each other (*D*′ = 0.72; *r* ^2^ = 0.23; *p* = 10^−15^). The B allele of eNOS 4 a/b polymorphism tended to be inherited with the T allele of −786 C/T, while the A allele of the former polymorphism was associated with the C allele of the latter in most cases.

## 4. Discussion

Many studies have been published about the influence of plasma eotaxin levels and variants of eotaxin gene on presence and severity of atherosclerosis but the results are controversial. Several authors reported an association of higher plasma levels with atherosclerotic burden [21, 22, 33]; on the other hand, other studies did not prove this [34, 35]. Higher eotaxin concentration was found in local blood samples from patients with acute coronary syndrome [36]. Among other effects, eotaxin was proved to stimulate migration of smooth muscle cells from media to intima of injured arterial wall [37]. This mechanism is considered to have an impact on atherosclerotic plaque progress and restenosis formation [38, 39]. Influence of eotaxin gene polymorphisms on cardiac events in Chinese diabetic patients was reported in the abovementioned paper [24]; the main result was increased morbidity in patients homozygote for the G allele of 67 G/A SNP in the first exon of the gene corresponding to alanine in resulting protein; this allele is associated with higher production and concentration of eotaxin [40, 41]. Likewise, in our previous study [30], there was greater susceptibility to acute coronary syndromes in subjects with homozygous 67 G/A GG genotype. This seemingly contradicts our current results, where GG genotype showed protective effect. However, given all subjects in our study suffered from CAD, our study group consisted of highly selected patients and their treatment may have been responsible for the results. Actually, the influence of A allele on prognosis can be explained by an effect of statin therapy. There is some evidence from both human patients and animal models of substantial chemokine levels reduction after statin treatment, including eotaxin [42–44]. Because A allele carriers have lower eotaxin levels [45], the protective effect of statin therapy may be reduced in comparison with GG homozygotes. This mechanism seems to be likely in our population of CAD patients, where the prevalence of statin therapy was very high (514/532, 96.6%).

In a large study with 14,916 American men, Zee et al. [46] studied the same polymorphism with different results. Authors compared data from 523 patients with myocardial infarction and 2092 persons who were free from cardiovascular disease during a follow-up (mean period 13.2 years). They reported that T23 (67 A) homozygotes had significantly higher risk of myocardial infarction: odds ratio (OR) 1.86; 95% confidence interval (CI), 1.15–3.01; *p* = 0.012; the effect remains significant even after adjusting for body mass index, history of hypertension, the presence of diabetes, and randomized treatment assignment. There was no significant difference between GG homozygotes and heterozygotes. Since there was no information about a treatment in that study, its impact cannot be excluded. The recessive model (AA versus GA + GG) in our study had relatively low power in all cases, there were only 16 AA homozygotes, to detect eventual risky effect of AA homozygote genotype. The linkage disequilibrium between 67 G/A and −384 A/G must be taken into account as well. Unlike the former one, the latter SNP was associated also with cardiac events and the “protective” and “risky” alleles were frequently inherited together.

The influence of genetic variants of different matrix metalloproteinase genes, such as MMP-9 [47, 48] or MMP-3 [49], on clinical outcome of CAD was described in previous studies. In our current study, no effect of rs640198 polymorphism in MMP-13 gene on either cardiac events or all-cause mortality was found both with and without adjustments. As no other study associating MMP-13 genetic variants and CAD exists so far, we can thus conclude that MMP-13 does not predict the prognosis of CAD, even when in our previous study based on the same population we found significant association of rs640198 with triple vessel disease. However, triple vessel disease itself was a significant predictor of both cardiac events and all-cause mortality. The gene-prognosis relation may be modified by commonly used treatment, as in the case of eotaxin.

The association of eNOS polymorphisms with CAD was reported in many studies. The large meta-analysis involving a total sample size of 69,235 subjects [50] confirmed the association of the three* NOS3* gene polymorphisms (Glu298Asp, −786 T/C, and 4 a/b) with the presence of CAD, but no significance was found for 4 a/b polymorphism in European population. No impact of −786 C/T or 4 a/b on CAD prognosis was found in our study.

In conclusion, a significant association between A allele in eotaxin 67 G/A single nucleotide polymorphism (SNP) in the first exon of the gene and lower survival rate in patients with coronary artery disease was found with and without adjustments for other factors. Similar results were observed for −384 A/G polymorphism in the same gene, which was also associated with cardiac events. Further studies will be needed to elucidate the mechanism of this effect.

## Figures and Tables

**Table 1:** Basic characteristics of patients. Data are shown as mean ± SD.

Total/men [*n*]	532/407
Age [years]	65.4 ± 9.6
BMI [kg/m^2^]	28.7 ± 4.0
One-vessel/two-vessel/three-vessel disease [*n*]	142/164/226
Smokers (%)	52%
Hypertension (%)	80%
Diabetes (%)	34%
EF (%)	55% (45%–60%)
Renal insufficiency: serum creatinine 105–200 *μ*mol/L (men) or 85–200 *μ*mol/L (women) (%)	23%
Creatinine [*μ*mol/L]	101 (92–113)
Uric acid [*μ*mol/L]	376 ± 106
Plasma glucose [mmol/L]	5.4 (4.9–6.5)
Cholesterol [mmol/L]	4.36 (4.72–5.10)
LDL [mmol/L]	2.38 (1.94–3.01)
HDL [mmol/L]	1.08 (0.93–1.27)
Triacylglycerols [mmol/L]	1.55 (1.20–2.09)
CRP [mg/L]	4.0 (1.8–7.4)
Medication (%):	
ASA	92%
Other antiplatelet	54%
Warfarin	3%
*β*-blocker	94%
ACE-inhibitor	65%
AT1-blocker	20%
Statin	97%
Other antilipids	3%

**Table 2:** Studied polymorphisms and genotype distribution (expressed as major allele homozygote/heterozygote/minor allele homozygote).

Polymorphism	rs	MAF (%)	*n*	Genotype distribution	HWE
MMP 13 G/T	rs640198	G>T; 0.32	470	220/198/52	yes
eNOS –786 C/T	rs2070744	T>C; 0.37	317	126/148/43	yes
eNOS 4 a/b	VNTR^*∗*^	b>a; 0.19	520	342/159/19	yes
Eotaxin –426 C/T	rs16969415	C>T; 0.07	445	384/56/5	yes
Eotaxin –384 A/G	rs17809012	A>G; 0.48	518	127/281/110	yes
Eotaxin 67 G/A	rs1129844	G>A; 0.15	532	391/125/16	yes

^*∗*^Variable number of tandem repeats.

**Table 3:** The preselection of candidate polymorphisms for death prediction based on log-rank *p* values; polymorphisms included in the Cox regression model construction are in ***bold italics***.

Polymorphism	*n*	Log-rank *p* (overall)	Log-rank *p* (dominant)	Log-rank *p* (recessive)
MMP 13 G/T	470	0.941	0.811	0.909
eNOS –786 C/T	317	0.200	0.129	0.720
***eNOS 4 a/b***	***520***	***0.458***	***0.025***	***0.419***
***Eotaxin –426 C/T***	***445***	***0.033***	***0.152***	***0.175***
***Eotaxin –384 A/G***	***518***	***0.084***	***0.533***	***0.050***
***Eotaxin 67 G/A***	***532***	***0.020***	***0.013***	***0.210***

**Table 4:** The preselection of candidate polymorphisms for cardiac events prediction based on log-rank *p* values. Polymorphisms included in the Cox regression model construction are in ***bold italics***.

Polymorphism	*n*	Log-rank *p* (overall)	Log-rank *p* (dominant)	Log-rank *p* (recessive)
MMP 13 G/T	470	0.677	0.710	0.526
eNOS –786 C/T	317	0.603	0.959	0.711
eNOS 4 a/b	520	0.937	0.910	0.642
Eotaxin –426 C/T	445	0.922	0.882	0.874
***Eotaxin –384 A/G***	***518***	***0.009***	***0.548***	***0.002***
Eotaxin 67 G/A	532	0.098	0.496	0.343

**Table 5:** Multivariate Cox regression, prediction of death by different models of inheritance.

Polymorphism	Model					
	Dominant		Recessive		Additive	
	HR (95% CI)	*p* value	HR (95% CI)	*p* value	HR (95% CI)	*p* value
eNOS 4 a/b	NS	NS	NS	NS	NS	NS
Eotaxin –426 C/T	NS	NS	NS	NS	NS	NS
Eotaxin –384 A/G	NS	NS	2.60 (1.15–5.87)	0.022	NS	NS
Eotaxin 67 G/A	3.36 (1.57–7.19)	0.0017	NS	NS	2.43 (1.39–4.26)	0.0019

**Table 6:** Multivariate Cox regression, prediction of cardiac endpoint by different models of inheritance.

Polymorphism	Model					
	Dominant		Recessive		Additive	
	HR (95% CI)	*p* value	HR (95% CI)	*p* value	HR (95% CI)	*p* value
Eotaxin −384 A/G	NS	NS	1.59 (1.20–2.10)	0.001	1.28 (1.07–1.54)	0.006

**Table 7:** Stepwise multivariate Cox regression model for survival.

Factor	HR (95% CI)	*p* value
Age (per 10 years)	2.32 (1.44–3.84)	0.0007
Smoking	3.84 (1.66–8.87)	0.002
Three vessel disease (versus one vessel disease)	3.89 (1.15–13.18)	0.029
Two vessel disease (versus one vessel disease)	1.32 (0.32–5.55)	0.701 (NS)
Eotaxin 67 G/A (GA+AA versus GG)	2.81 (1.35–5.85)	0.006
